# Epigenetics in rheumatic diseases

**DOI:** 10.1186/1546-0096-12-S1-I12

**Published:** 2014-09-17

**Authors:** Steffen Gay

**Affiliations:** 1 Rheumatology, University Hospital, Zürich, Switzerland

## 

With enormous speed novel data are emerging about regulating the expression of the genetically encoded information [1]. This highly complex regulatory network called *epigenetics* includes acetylation, methylation, phosphorylation, sumoylation and non-coding RNAs (ncRNA), such as miRNA and lncRNAs. Our laboratory is addressing over the past decade inflammatory rheumatic diseases [2], like rheumatoid arthritis (RA), AS, SSc and pulmonary hypertension and thereby searching for the regulation of pro-inflammatory cytokines [3,4], novel diagnostic signatures and new therapeutic targets. In this regard, DNA demethylation of RA synovial cells can be modulated by targeting specific enzymes [5]. Also, miRNA signatures for new response markers are in development [6].

## Disclosure of interest

None declared.
